# Higher prevalence of multidrug-resistant extended-spectrum β-lactamases producing *Escherichia coli* in unorganized pig farms compared to organized pig farms in Mizoram, India

**DOI:** 10.14202/vetworld.2020.2752-2758

**Published:** 2020-12-23

**Authors:** R. Mandakini, P. Roychoudhury, P. K. Subudhi, H. Kylla, I. Samanta, S. Bandyopadhayay, T. K. Dutta

**Affiliations:** 1 Department of Veterinary Microbiology, College of Veterinary Sciences and Animal Husbandry, Central Agricultural University, Aizawl, Mizoram, India; 2 Department of Veterinary Microbiology, West Bengal University of Animal and Fishery Sciences, Kolkata, West Bengal, India; 3 ICAR-Eastern Regional Station of IVRI, Kolkata, West Bengal, India

**Keywords:** *Escherichia coli*, extended-spectrum β-lactamases, India, Mizoram, multidrug-resistant, pig

## Abstract

**Aim:**

The present study was conducted to record the prevalence of multidrug-resistant (MDR), extended-spectrum β-lactamases (ESBLs) producing *Escherichia coli* from pig population of organized and unorganized farms of Mizoram and to record the presence of ESBLs, non-ESBLs, and integrons.

**Materials and Methods:**

Fecal samples were collected from pigs under organized (n=40) and unorganized (n=58) farms of Mizoram. Samples were processed for isolation and identification of *E. coli* by conventional techniques, BD Phoenix™ automated bacterial system, and polymerase chain reaction (PCR) based confirmatory test. All the isolates were subjected to antimicrobial sensitivity test by disk diffusion assay and ESBLs production by double-disk synergy test (DDST). The ESBLs producing isolates were subjected to PCR for determination of ESBLs genes and all the isolates were screened for non-ESBLs genes and integrons by PCR.

**Results:**

A total of 258 *E. coli* was isolated and identified from organized (n=120) and unorganized farms (n=138). Majority of the *E. coli* isolates exhibited high level of resistance against amoxicillin (Ax) (81.78%), cefalexin (85.42%), co-trimoxazole (50.78%), sulfafurazole (69.38%), tetracycline (65.89%), and trimethoprim (TR) (51.94%). Statistically highly significant (p<0.01) variations in resistance among the isolates from organized and unorganized farms were recorded in case of Ax, ampicillin, cephalexin, ciprofloxacin, co-trimoxazole, gentamicin, piperacillin, and TR. By DDST, 65.89% isolates were recorded as ESBLs producer, of which 82/120 (68.33%) and 88/138 (63.77%) were from organized and unorganized farms, respectively. A total of 29/258 (11.24%) isolates were positive for at least one ESBLs gene. *bla_TEM_* was most frequently (9.69%) gene, followed by *bla_CTX_*-M (5.04%) and *bla_CMY_* (0.78%). Altogether, 6 (5.00%), 4 (3.33%), and 2 (1.67%) isolates from the organized farms were positive for *bla_CTX-M_*, *bla_TEM_*, and *bla_CMY_* genes, respectively. Similarly, 21 (15.22%) and 7 (5.07%) isolates from the unorganized farms were positive for *bla_TEM_* and *bla_CTX-M_* genes, respectively. None of them were positive for *bla_SHV_* genes. Altogether 57 (22.09%), 9 (3.49%), 66 (25.58%), 78 (30.23%), 21 (8.14%), and 18 (6.98%) isolates were positive for *tetA*, *tetB*, *sul1*, *sul2*, *aadA*, and *dfrla* genes, respectively. The prevalence of non-ESBLs genes was higher in the *E. coli* isolates from the unorganized farms than organized farms.

**Conclusion:**

MDR and ESBLs producing *E. coli* are circulating among the pigs and their environment in Mizoram. Pigs under unorganized farms exhibited higher level of resistance against majority of the antimicrobials, including third-generation cephalosporins, which might be an indication of overuse or misuse of antibiotics under the unorganized piggery sectors in Mizoram.

## Introduction

*Escherichia coli* is a normal gastrointestinal microflora of the worm-blooded animals, including human beings, which are also capable to develop various clinical conditions under favorable environment. To reduce the morbidity and mortality of the disease conditions associated with bacterial infections, application of antibiotics is common practice since its discovery. However, *E. coli* is well known to be capable of the development of antimicrobial resistance (AMR) very frequently through accepting and transferring the resistance traits under selection pressure [1]. Development and dissemination of resistance traits from the resistant *E. coli* to other groups of bacteria are multifaceted and are probably evolving constantly. Reports on multidrug-resistant (MDR) bacteria in human [2], animals [3], or environment [4] are well documented throughout the world by various workers. Development, maintenance, and dissemination of AMR among the bacteria in human and animals in any particular geographical region are a dynamic process and depend on multiple factors [5]. Excessive use and abuse of antimicrobials are considered to be the most important driving factors, particularly in the developing and underdeveloped countries [6].

Resistance to antimicrobial agents in microorganisms can either be intrinsic or acquired. Intrinsic resistance comprises all of the inherent properties, which are located on the chromosome of a particular species. *E. coli* naturally produces a chromosomal non-inducible AmpC ß-lactamase expressed at a low level, which is responsible for resistance to penicillin [7]. Other chromosomal resistance mechanisms include low permeability for a specific drug and/or due to the intrinsic presence of multidrug efflux pumps [8]. Most common type is acquired resistance, in which a strain of an originally susceptible species becomes resistant. Acquired resistance mechanisms involve mutations in genes targeted by the antimicrobial agent or transfer of resistance determinants borne on plasmids, bacteriophages, transposons, and other mobile genetic elements [1].

Among the worldwide array of antibiotics, β-lactams are the most widely used agents. The most common cause of resistance to β-lactam antibiotics are the production of β-lactamases. Over the years, many β-lactam antibiotics have been developed; however, with each new class of antibiotics, a new β-lactamase emerged that caused resistance to that class of drug [9]. Extended-spectrum β-lactamases (ESBLs) are the rapidly evolving group of β-lactamase enzymes produced by the Gram-negative bacteria, which have the ability to hydrolyze all cephalosporins and aztreonam but are inhibited by clavulanic acid [4]. In addition to the ESBLs production, coresistance against other antimicrobial agents such as sulfa, carbapenems, aminoglycosides, and tetracyclines (TE) are also reported among the *E. coli* isolates by various workers [10].

In Mizoram, pig farming is the most important backbone of the rural economy. On most occasions, pigs are reared by individual farmers under swill feeding to minimize the input costs. However, few organized farms are also available for large scale production of pork to meet the local demands. So far, no systematic study was conducted to find out the existence of MDR *E. coli* in the organized and unorganized farming system in Mizoram. Sporadic studies conducted in North Eastern India confirmed the presence of ESBLs (*bla_CTX-M_* and *bla_SHV_*) through isolation of *E. coli* from fecal samples of pigs [11]. An ESBL-producing *E. coli* possessing *bla_CTX-M_* and *bla_SHV_* associated with human diarrhea has been reported in India [12]. Puii *et al*. [4] also reported the prevalence of ESBL producing genes (*bla_TEM_*, *bla_CTX-M_*, and *bla_CMY_*) in the enteric bacteria of pig population in Northeastern India.

Although few sporadic reports are available, no systematic efforts have been made till date to detect the MDR *E. coli* in pigs maintained under organized and unorganized farming system of Mizoram and their subsequent characterization. The present study was formulated on isolation, identification, drug resistance properties, and molecular characterization of MDR *E. coli* strains prevalent in pigs maintained under various farming system.

## Materials and Methods

### Ethical approval

As there were no invasive techniques applied under any of the experiments, including sample collections, ethical approval is not applicable for this study.

### Sample collection

Fresh fecal samples were collected randomly from pigs of Mizoram during the period of September 2012 to May 2014. A total of 98 samples were collected randomly from 40 pigs maintained under organized (n=4) and 58 pigs from unorganized (n=29) farming system irrespective of age, sex, and with or without a history of diarrhea during the study. All the samples were collected using a sterilized absorbent cotton swab. However, for collection of samples from distant locations, sterilized swabs dipped in brain heart infusion broth were used as a transport medium and transported to the laboratory under cold chain (4°C) for further processing.

### Isolation and identification of *E. coli*

The collected fecal samples were processed for isolation and identification of *E. coli* using standard bacteriological techniques. Five single pure colonies were picked from each inoculated plate for further confirmation. All the isolates were further confirmed by BD Phoenix™ automated bacterial identification system and *E. coli* species-specific polymerase chain reaction (PCR) targeting the *uidA* gene. All the isolates were stored as pure culture in semi-solid agar at 4°C as well as in Luria Bertani (LB) broth (HiMedia, Mumbai) containing 25 % glycerol (v/v) (Sigma) at −80°C for further use.

### Antibiotic susceptibility test and confirmatory test for ESBLs

Antimicrobial susceptibility test was done on Mueller-Hinton agar (HiMedia, Mumbai) plate as per the recommendation of Clinical Laboratory Standard Institute [13] using the following commercially (HiMedia, Mumbai) available antibiotic disks: Amoxicillin (Ax, 30 mcg), ampicillin (AMP, 10 mcg), aztreonam (AZ, 30 mcg), cefalexin (CN, 30 mcg), cefixime (CFM, 30 mcg), cefotaxime (CTX, 30 mcg), ceftazidime (CAZ, 30 mcg), ceftriaxone (CTR, 30 mcg), ciprofloxacin (CIP, 5 mcg), co-trimoxazole (COT, 1.25/23.75 mcg), gentamicin (GEN, 10 mcg), imipenem (IPM, 10 mcg), nalidixic acid (NA, 30 mcg), piperacillin (PI, 100 mcg), streptomycin (S, 10 mcg), sulfafurazole/sulfisoxazole (SF, 300 mcg), TE (TE, 30 mcg), and trimethoprim (TR, 30 mcg) (Table-1).

**Table-1:** Details of the oligonucleotide primers used in the present study.

Primer name	Sequence (5’ → 3’)	Expected amplicon size (bp)	Annealing temperature (°C)
*bla_TEM_*	ATAAAATTCTTGAAGACGAAA GACAGTTACCAATGCTTAATC	1080	53
*bla_SHV_*	CTTTCCCATGATGAGCACCT CGCTGTTATCGCTCATGGTA	206	60
*bla_CTX-M_*	CAATGTGCAGCACCAGTAA CGCGATATCGTTGGTGGTG	540	58
*bla_CMY_*	TGGCCAGAACTGACAGGCAAA TTTCTCCTGAACGTGGCTGGC	462	60
*tetA*	GTAATTCTGAGCACTGTCGC CTGCCTGGACAACATTGCTT	937	57
*tetB*	TTGGTTAGGGGCAAGTTT GTAATGGGCCAATAACAC	659	57
*aadA*	GCAGCGCAATGACATTCTTG ATCCTTCGGCGCGATTTTG	282	58
*DfrIa*	GTGAAACTATCACTAATGG TTAACCCTTTTGCCAGATTT	474	53
*IntI1*	GGGTCAAGGATCTGGATTTCG ACATGGGTGTAAATCATCGTC	483	60
*IntI2*	CACGGATATGCGACAAAAAGGT GTAGCAAACGAGTGACGAAATG	788	60
5’-CS	GGCATACAAGCAGCAAGC	variable	52
3’-CS	AAGCAGACTTGACCTGAT		
Ti-F	ACCTTTTTGTCGCATATCCGTG	variable	55
Ti-B	CTAACGCTTGAGTTAAGCC		

Confirmatory test for ESBLs production was carried out using CTX (30 mcg), Ax (30 mcg), and CAZ (30 mcg) alone as well as CTX/clavulanate (30/10 mcg), Ax/clavulanate (30/10 mcg), and CAZ/clavulanate (30/10 mcg) combination as per the recommendation of CLSI [13]. Both the disks were placed at least 25 mm apart, center to center, on a lawn culture of the test isolate in Mueller-Hinton agar plate and incubated overnight at 37°C. The difference in zone diameters with and without clavulanic acid was measured. When there was an increase of ≥ 5mm in inhibition zone diameter around antimicrobial agent tested in combination with clavulanic acid versus its inhibition diameter zone, when tested alone was confirmed as potent ESBLs producing isolates.

### Genotypic detection of antibiotic resistance profile

The presence of AMR genes was analyzed by PCR using bacterial lysate as a template DNA. Detection of AMR genes were performed for sulfamethoxazole resistant isolates (*Sul* and *Sul2*), AMP resistance isolates (*bla_TEM_, bla_SHV_, bla_CTX-M_*, and *bla_CMY_*), TE resistant isolates (*tetA, tetB*), S resistant isolates (*aadA*), and TR resistant isolates (*dfrIa*) using specific oligonucleotide primers. Details of oligonucleotide primers are depicted in Table-1. PCR conditions were followed as per the method described by Mandakini *et al*. [14] with suitable modifications. The repeatability of the assay was checked by repeating the PCR 3 times.

### Detection of antibiotic resistance integrons

The MDR isolates were also screened for the presence of class 1 and 2 integrons, namely, *intI1* and *intI2* as well as its gene cassettes 5CS/3CS and TiB/TiF. PCR amplification was used to detect Class 1 and Class 2 integrase genes (*intI1* and *intI2*, respectively) using specific primers. The condition used for amplification of the two integrase genes was as follows: Initial denaturation at 94°C for 5 min, 30 amplification cycles consisting of 50 s at 94°C, 50 s at 60°C and 1 min at 72°C, and final extension for 6 min. Amplification of variable region of Class 1 and Class 2 integrons was performed using the primers 5’-CS/3’-CS and Ti-F/Ti-B, as per the conditions and procedures described previously by Mandakini *et al*. [14].

## Results

### Isolation of *E. coli*

A total of 258 *E. coli* was isolated and identified by traditional bacteriological techniques, BD Phoenix automated bacterial system and PCR based confirmation, of which 120 and 138 were from organized and unorganized farms, respectively.

### Antibiotic susceptibility of the isolates

Majority of the *E. coli* isolates from organized and unorganized farms of Mizoram exhibited high level of resistance against Ax (81.78%), CN (85.42%), co-trixamozole (50.78%), SF (69.38%), TE (65.89%), and TR (51.94%). IPM was found to be the most sensitive antimicrobials. Among the *E. coli* isolates from organized farms, highest resistance was recorded against CN (96.00%), followed by AMP (92.50%), SF (64.17%), and TE (61.67%). Similarly, in case of *E. coli* isolates from unorganized farms maximum resistance was recorded against CN (73.91%) and SF (73.91%), followed by AMP (72.46%), TE (69.57%), and TR (64.49%). *E. coli* isolates from organized farms showed higher resistance against Ax, cephalexin, cefixime, and NAs than their unorganized counterparts. On the other hand, *E. coli* isolates from unorganized farms showed higher resistance against AMP, CIP, co-trimoxazole, PI, SF, and TR. Statistically highly significant (p<0.01) variations in resistance amongst the isolates from organized and unorganized farms were recorded in case of Ax, AMP, cephalexin, CIP, co-trimoxazole, gentamicin, PI, and TR (Table-2).

**Table-2:** Antibiotic resistance profile of *E. coli* isolated from fecal samples of pig from organized and unorganized farms of Mizoram, India.

Antibiotics	Organized farm (n=120)	Unorganized farm (n=138)	Total (n=138)	ND value
Amoxicillin	111 (92.50)	100 (72.46)	211 (81.78)	4.16**
Ampicillin	27 (22.50)	61 (47.66)	88 (35.48)	3.67**
Aztreonam	18 (16.36)	31 (22.46)	49 (19.76)	1.52^NS^
Cephalexin	144 (96.00)	102 (73.91)	246 (85.42)	4.58**
Cefixime	48 (40.00)	38 (27.54)	86 (33.33)	2.12*
Cefotaxime	17 (14.17)	24 (17.39)	41 (15.89)	0.71^NS^
Ceftazidime	35 (29.17)	40 (28.99)	75 (29.07)	0.03^NS^
Ceftriaxone	12 (10.00)	20 (14.49)	32 (12.40)	1.09^NS^
Ciprofloxacin	3 (2.50)	18 (13.04)	21 (8.14)	3.09**
Co-trimoxazole	42 (35.00)	89 (64.49)	131 (50.78)	4.73**
Gentamicin	7 (5.83)	24 (17.39)	31 (12.02)	2.85**
Imipenem	0 (0.00)	2(1.45)	2 (0.78)	1.32^NS^
Nalidixic acid	46 (38.33)	33 (23.91)	79 (30.62)	2.51*
Piperacillin	32 (26.67)	63 (45.65)	95 (36.82)	3.15**
Streptomycin	8 (6.67)	20 (14.49)	28 (10.85)	2.02*
Sulfafurazole	77 (64.17)	102 (73.91)	179 (69.38)	1.69^NS^
Tetracycline	74 (61.67)	96 (69.57)	170 (65.89)	1.33^NS^
Trimethoprim	45 (37.50)	89 (64.49)	134 (51.94)	4.33**

* Statistically significant (p<0.05) between organized and unorganized farm;

** Highly significant (p<0.01) between organized and unorganized farm; ^NS^Not significantly different (p<0.01) between organized and unorganized farm. Figures in parentheses are indicated as percentages.

By double-disk synergy test method, a total of 170/258 (65.89%) *E. coli* isolates were recorded as ESBLs producer, of which 82/120 (68.33%) and 88/138 (63.77%) were from organized and unorganized farms, respectively.

### Genotypic detection of β-lactamase genes

A total of 29/258 (11.24%) *E. coli* isolates from Mizoram were found to be positive for at least one ESBLs gene. *bla_TEM_* was the most frequently (25/258; 9.69%) gene, followed by *bla_CTX_*-M (13/258; 5.04%) and *bla_CMY_* (2/258; 0.78%). *bla_SHV_* gene could not be detected in any of the isolates under the present study. Altogether, 6/120 (5.00%), 4/120 (3.33%), and 2/120 (1.67%) *E. coli* isolates from the organized farms were positive for *bla_CTX-M_*, *bla_TEM_*, and *bla_CMY_* genes, respectively. Similarly, 21/138 (15.22%) and 7/138 (5.07%) *E. coli* isolates recovered from the unorganized farms were positive for *bla_TEM_* and *bla_CTX-M_* genes, respectively, and none of them were positive for *bla_CMY_* and *bla_SHV_* genes (Table-3).

**Table-3:** Distribution of antimicrobial resistance genes among *E. coli* isolated from fecal samples of pigs from different organized and unorganized farms of Mizoram, India.

*E. coli* isolate detail	*bla_TEM_*	*bla_CTX-M_*	*bla_CMY_*	*bla_SHV_*	*tetA*	*tetB*	*sul1*	*sul2*	*aadA*	*dfrIa*	*IntI1*
Organized farms (n=120)	4 (3.33)	6 (5.00)	2 (1.67)	-	12 (10.00)	-	6 (5.00)	24 (20.00)	12 (10.00)	-	6 (5.00)
Unorganized farms (n=138)	21 (15.22)	7 (5.07)	-	-	45 (32.60)	9 (6.52)	60 (47.38)	54 (39.13)	9 (6.52)	18 (13.04)	18 (13.04)
Total (n=258)	25 (9.68)	13 (5.04)	2 (0.78)	-	57 (22.09)	9 (3.49)	66 (25.58)	78 (30.23)	21 (8.14)	18 (6.98)	24 (9.30)

Figures in parentheses are indicated as percentages

### Genotypic characterization of non-ESBLs genes

In Mizoram, of the 258 isolates from both organized and unorganized farms, a total of 83/258 (32.17%) *E. coli* isolates were positive for at least one non-ESBLs genes, of which 57/258 (22.09%), 9/258 (3.49%), 66/258 (25.58%), 78/258 (30.23%), 21/258 (8.14%), and 18/258 (6.98%) *E. coli* isolates were found to be positive for *tetA*, *tetB*, *sul1*, *sul2*, *aadA*, and *dfrla* genes, respectively (Table-3). The prevalence of non-ESBLs genes was more in the *E. coli* isolates from the unorganized farms compared to the isolates from organized farms. *tetB* and *dfrIa* genes were not found in any *E. coli* isolates from the organized farms (Table-3).

### Detection of antibiotic resistance integrons and gene cassettes

In the present study, a total of 24/258 (9.30%) *E. coli* isolates were found to be positive for Class 1 integrons (*intI1)*, of which 6/120 (5.00%) were from organized farms and 18/138 (13.04%) were from unorganized farms (Table-3).

## Discussion

So far, a very limited study has been conducted on the prevalence of MDR *E. coli* in pigs under organized and unorganized farming set up in Northeastern region of India except few sporadic reports on ESBLs producing enteric bacteria in man and animals. The present study was conducted to investigate the prevalence of MDR *E. coli* isolates from pigs of organized and unorganized farms in Mizoram with special emphasis on determination of ESBLs production, determination of ESBLs and non-ESBLs genes, as well as integrons and gene cassettes.

In the present study, a total of 258 *E. coli* were isolated and identified from 98 fecal samples collected from pigs of organized and unorganized farms of Mizoram, India with or without a history of diarrhea. *E. coli* is a commensal in the intestinal tract of man and animals. The population in the gut may vary depending on the physiological and/or pathological status of the host. A similar or little variable rate of *E. coli* isolation from various animals is reported from India and abroad. Earlier, from the same laboratory, Lalzampuia *et al*. [11] isolated 102 *E. coli* from 53 fecal samples from pigs in Mizoram. On the other hand, Lalruatdiki *et al*. [15] could recover 867 *E. coli* from 228 fecal samples from pigs of Meghalaya and Assam. On the other hand, Samanta *et al*. [16] reported only 76 *E. coli* from 200 fecal samples in West Bengal, India. The rate of isolations of *E. coli* from fecal samples may vary depending on the nature of samples, types of media used, laboratory practices of the researchers, treatment status of the host, and so on. A number of *E. coli* colonies picked up from a particular sample is also variable. It may lead to variation in the frequency of *E. coli* isolates from a particular number of samples. In this study, we have picked up a minimum five suspected colonies randomly from primary culture plate for further confirmation by morphological and biochemical characteristics to avoid false-negative result.

In the present study, 62/258 (24.03%) *E. coli* isolates exhibited resistance to antimicrobial agents against the minimum of three classes, hence, MDR. The resistance pattern of *E. coli* isolates against the third-generation cephalosporins was variable. Moreover, variations were also recorded between the isolates from organized and unorganized farms. NARMS report showed that resistance to CTR ranged from 6.3% to 13.5% among *E. coli* isolated from chickens during 2000-2008. In contrast to our result, Sasirekha *et al*. [17] reported 84%, 75%, and 85% resistance to CTX, CTR, and CAZ, respectively, among the *E. coli* isolates from pigs. On the other hand, Rosengren *et al*. [18] reported no resistant isolates against CTR and <1% resistance to cefoxitin and ceftiofur. Interestingly, *E. coli* isolates from unorganized farms exhibited higher resistance against majority of the commonly used antimicrobials in Mizoram. It may be due to overuse of antibiotics through self-medication, introduction of antimicrobial residues through swill feeding or even through environmental contamination, particularly contaminated water [4]. Most of the ESBLs producing organisms were also co-resistant to fluoroquinolones, aminoglycosides, and COT, which corroborates with the earlier findings [14], where the ESBLs producing enteric bacteria were found to be resistant to other groups of antibiotics including aminoglycosides, TE, sulfonamides, TR, and chloramphenicol. Development of co-resistance against other antibiotics along with β-lactam antibiotics by the ESBLs producing organisms generally appears in the large plasmids, where most of the resistance genes may coexist [1].

In this study, 6/120 (5.00%), 4/120 (3.33%), and 2/120 (1.67%) *E. coli* isolates from the organized farms were positive for *bla_CTX-M_*, *bla_TEM_*, and *bla_CMY_* genes, respectively. However, on the other hand, 21/138 (15.22%) and 7/138 (5.07%) *E. coli* isolates from the unorganized farms were positive for *bla_TEM_* and *bla_CTX-M_* genes, respectively. In addition, none of them were positive for *bla_CMY_* and *bla_SHV_* genes (Table-2), which is in corroboration with the observation of other workers from abroad except one report from the same laboratory [11], wherein a small scale study, 7(5.07%) and 3(2.17%) *E. coli* isolates from pigs of Mizoram were recorded as positive for *bla_CTX-M-1_* and *bla_TEM_* gene, respectively, of which 3 (2.17%) isolates were positive for both the genes. Globally, the prevalence of *CTX-M* producing *E. coli* is varied between 0.8% in Europe to 25.0% [19]. The variation of results, in this study, in comparison to the other reports, may be due to the lower expression of *CTX-M* genes. *bla_CTX-M_* genes spread throughout the community, mostly through the transmission of plasmids, and some studies have also reported that animals may serve as a possible source for the dissemination of ESBL-encoding genes to humans [12]. Indeed, from the same laboratory *bla_CTX-M_* genes in *E. coli* from human and animals from Mizoram is already published [11,12], which also suggest that the transfer of extended-spectrum cephalosporin resistance between animals and humans frequently occur [4]. In the present study, the TE (*tetA* and *tetB*) and sulfonamides (*sul1* and *sul2*) resistance genes were detected most frequently in unorganized farms. The present findings are in contrast with the earlier findings in Arunachal Pradesh, where non-ESBLs associated genes were more from the isolates from the organized farms [14]. It might be due to more frequent use of such classes of antimicrobials under unorganized farming system in recent years. The presence of *tet* and *sul* genes has been reported as a prevailing mechanism for TE and sulfonamide resistance, respectively, in *E. coli* isolates from pet animals and wild animals. As mentioned in an earlier section that in Mizoram, the pigs under unorganized farms are getting exposed to more antimicrobials compared to its organized counterparts. Class 1 integrons are the most common antibiotic-resistant genes found in the clinical isolates of Gram-negative bacteria [14]. In an earlier, Kar *et al*. [10] also reported class integrons in clinical isolates of *E. coli* from Odisha, India. Integrons have been identified as a primary source of resistance genes and are claimed to be reservoirs of AMR genes within microbial populations.

## Conclusion

In the present study, MDR *E. coli* were isolated and identified from the pigs under organized and unorganized farming system in Mizoram, India. *E. coli* isolates from unorganized farms exhibited significantly higher resistance against the third-generation cephalosporins and AMP. In addition, the same isolates were also exhibited more ESBLs and non-ESBLs AMR genes, which correlate with the phenotypic observations. The presence of more number of MDR *E. coli* isolates in unorganized farming set up compared to the organized pig farms is a serious indication of misuse or overuse of antimicrobials in this environment.

## Authors’ Contributions

RM: Collection of samples, processing for isolation and identification of bacteria, AST, PCR assays. TKD: Genesis of concept of the work and preparation of the manuscript. PR: Detection of ESBLs genes by PCR assays. PKS: AMR sensitivity assay and data analysis. HK: Sample collection and processing for isolation and identification. IS: Preparation of manuscript and editing. SB: Preparation of manuscript and editing. All authors read and approved the final manuscript.

## Competing Interests

The authors declare that they have no competing interests.

## Publisher’s Note

Veterinary World remains neutral with regard to jurisdictional claims in published institutional affiliation.
